# SNP association study in PMS2-associated Lynch syndrome

**DOI:** 10.1007/s10689-017-0061-3

**Published:** 2017-11-17

**Authors:** Sanne W. ten Broeke, Fadwa A. Elsayed, Lisa Pagan, Maran J. W. Olderode-Berends, Encarna Gomez Garcia, Hans J. P. Gille, Liselot P. van Hest, Tom G. W. Letteboer, Lizet E. van der Kolk, Arjen R. Mensenkamp, Theo A. van Os, Liesbeth Spruijt, Bert J. W. Redeker, Manon Suerink, Yvonne J. Vos, Anja Wagner, Juul T. Wijnen, E. W. Steyerberg, Carli M. J. Tops, Tom van Wezel, Maartje Nielsen

**Affiliations:** 1 Department of Clinical Genetics, Leiden University Medical Centre, Albinusdreef 2, 2333 ZA Leiden, The Netherlands; 2 Department of Pathology, Leiden University Medical Centre, Leiden, The Netherlands; 3 Department of Clinical Genetics, University Medical Centre Groningen, Groningen, The Netherlands; 4 Department of Clinical Genetics, Maastricht University Medical Centre, Maastricht, The Netherlands; 5 Department of Clinical Genetics, VU Amsterdam, Amsterdam, The Netherlands; 6 Department of Clinical Genetics, University Medical Centre Utrecht, Utrecht, The Netherlands; 7 Department of Clinical Genetics, The Netherlands Cancer Institute, Amsterdam, The Netherlands; 8 Department of Clinical Genetics, Radboud University Medical Centre, Nijmegen, The Netherlands; 9 Department of Clinical Genetics, Academic Medical Centre, Amsterdam, The Netherlands; 10 Department of Clinical Genetics, Erasmus Medical Centre, Rotterdam, The Netherlands; 11 Department of Medical Statistics, Leiden University Medical Centre, Leiden, The Netherlands

**Keywords:** Lynch syndrome, PMS2, SNP, Cancer risk, Colorectal cancer, Modifiers

## Abstract

Lynch syndrome (LS) patients are at high risk of developing colorectal cancer (CRC). Phenotypic variability might in part be explained by common susceptibility loci identified in Genome Wide Association Studies (GWAS). Previous studies focused mostly on *MLH1, MSH2* and *MSH6* carriers, with conflicting results. We aimed to determine the role of GWAS SNPs in *PMS2* mutation carriers. A cohort study was performed in 507 *PMS2* carriers (124 CRC cases), genotyped for 24 GWAS SNPs, including SNPs at 11q23.1 and 8q23.3. Hazard ratios (HRs) were calculated using a weighted Cox regression analysis to correct for ascertainment bias. Discrimination was assessed with a concordance statistic in a bootstrap cross-validation procedure. Individual SNPs only had non-significant associations with CRC occurrence with HRs lower than 2, although male carriers of allele A at rs1321311 (6p21.31) may have increased risk of CRC (HR = 2.1, 95% CI 1.2–3.0). A polygenic risk score (PRS) based on 24 HRs had an HR of 2.6 (95% CI 1.5–4.6) for the highest compared to the lowest quartile, but had no discriminative ability (c statistic 0.52). Previously suggested SNPs do not modify CRC risk in *PMS2* carriers. Future large studies are needed for improved risk stratification among Lynch syndrome patients.

**Electronic supplementary material:**

The online version of this article (10.1007/s10689-017-0061-3) contains supplementary material, which is available to authorized users.

## Introduction

Lynch syndrome (LS) accounts for 2–4% of all CRCs and is characterized by a high risk for developing malignancies, most notably colorectal cancer (CRC) and endometrial cancer (EC). The underlying cause is a germline mutation in one of the mismatch repair (MMR) genes: *MLH1, MSH2* (*EPCAM*), *MSH6* or *PMS2*. Mutations in all MMR genes are associated with a significantly increased cancer risk compared to the general population, although *MSH6* and *PMS2* carriers show lower penetrance compared to *MLH1* and *MSH2* carriers [1–3]. Within and between family variability is commonly observed and a range of theories have been proposed to explain the phenomenon, such as genotype–phenotype correlations, parent-of-origin effects, lifestyle factors and the influence of common susceptibility loci. The latter, mainly single nucleotide polymorphisms (SNPs), were identified in genome wide association studies (GWAS) in large cohorts consisting of sporadic CRC cases [4]. Among these candidate SNPs, previous studies have identified statistically significant effects of multiple SNPs in LS patients, and independent studies replicated the effect of SNPs rs3802842 (11q23.1) and rs16892766 (8q23.3) among *MLH1* carriers [5, 6]. It should be noted, however, that others have failed to replicate these findings [7, 8]. Although the latter studies analyzed cohorts of similar size to our own, few or no *PMS2* carriers were included [7]. Due to a relatively low penetrance and high phenotypic variability, this specific subset of LS patients might be of particular interest [9]. In a previous study among 377 *PMS2* carriers, we found age at CRC diagnosis to vary widely (range 26–86 years) and mean age of index carriers and mutation-positive family members differed by 10 years [3]. In the current study, we aim to determine whether these SNPs modify CRC risk in a large cohort of *PMS2* mutation carriers.

## Materials and methods

### Sample collection

DNA extracted from leucocyte DNA was collected from 8 Dutch family cancer clinics. Index carriers included in this study were sent in between 2007 and 2016 to the Clinical Genetics department, because of a clinical suspicion of LS, e.g. LS-associated cancer at a young age and/or a positive family history. Mutation analysis was initiated based on the presence of histological hallmarks [microsatellite instability (MSI) or loss of PMS2 expression in the tumor] and/or when the family complied with the Bethesda Criteria [10]. Participating clinics provided DNA samples and clinical data on CRC, age at diagnosis, other cancer development and polypectomy. Controls were defined as carriers that were tested pre-symptomatically, after a pathogenic mutation was identified in the index carrier of the family. All carriers are referred to gastroenterology departments after the diagnosis has been established, which then adhere to international surveillance guidelines, i.e. colonoscopies every 1–2 years from 25 years of age [11]. Data was analyzed anonymously. The study was approved by the medical ethical committee of Leiden University Medical Centre, protocol ID P01-019.

### Genotyping

*PMS2* genotyping in this cohort was carried out as previously described [3]. SNP genotyping was done at the LUMC laboratory using a KASp genotyping assay (LGC Genomics, Hoddesdon, UK). Primers were designed using Primerpicker (KBioscience, Hoddesdon, UK) and are available upon request. All oligonucleotides were obtained from Eurofins Genomics (Ebersberg, Germany). Genotypes were called using the CFX manager software v3.0 (Bio-Rad, Veenendaal, the Netherlands).

### Statistical analysis

*PMS2* carriers were analyzed as a birth cohort. A Cox-proportional hazards regression model was fitted to estimate hazard ratios (HRs), with age at CRC as endpoint and SNP genotype as independent variable. Patients without CRC were censored at the last age known to be alive. The HR was calculated separately for heterozygous and homozygous carriers of the risk allele, with homozygotes of the non-risk allele as reference category. We also calculated the per allele HR (additive model). Cox-regression analyses were also stratified for gender. These sub-analyses only includes a per allele (additive) model, due to multiple testing risks. Missing age at CRC diagnosis (n = 3) was imputed using median age of CRC in the general population (age 70, n = 2) or set at 1 year before death (n = 1). The proportional hazards assumption was investigated by examining the scaled Schoenfeld residuals with a formal statistical test and by visual inspection.

Previous studies have described the oversampling of cases in clinic-based cohorts. Moreover, affected family members are more likely to be tested for the mutation and this too results in oversampling of cases. To adjust for this non-random sampling, we used a weighted cohort approach as previously described [12]. Standard errors were corrected for familial clustering of risk by using the Huber–White sandwich estimator [13].

We also calculated two polygenic risk scores (PRS) [14] based on (1) the odds ratios (ORs) reported in the meta-analysis by Ma et al. (Supplemental Table 1) and (2) based on our HRs from the current study [4]. Kaplan Meier (KM) and Cox regression analysis were concurrently performed. A concordance statistic was calculated to assess the discriminative value of the 24 SNP model. The optimism in the concordance statistic was estimated by fitting the model in each of 500 bootstrap samples (drawn with replacement), and validation in the original sample. Analyses were initially performed for patients with complete data. We also performed imputation of missing values based on the correlation structure between SNPs and with the outcome (transcan function in R software, version 3.2). Since results were similar, we only present complete case results.

Lastly, a post hoc power analysis was performed to assess the chance of finding significantly increased risks using the collected cohort, which contains all currently known *PMS2* mutation carriers in the participating centers (Supplemental Fig. 1). We had at least 80% power to find an HR of 1.5 for the majority of SNPs. A more detailed description of the statistical analysis is available in the supplementary methods (Supplemental File 1). Statistical analyses were performed using STATA version 14 (StataCorp. 2015. Stata Statistical Software: Release 14. College Station, TX: StataCorp LP) and R software (version 3.2, using the rms library).

## Results

### Cohort

In total, 521 samples from carriers with a germline *PMS2* mutation were genotyped, derived from 152 families. Fourteen patients were excluded because (1) they were younger than 25 years at censoring and were therefore not yet at risk of developing CRC (n = 11) or (2) insufficient clinical data was available (n = 3, including one CRC case). The analyzed cohort consisted of 124 cases (*PMS2* carriers with CRC) and 383 controls (*PMS2* carriers without CRC), with attributed person years of 6527 and 19,549, respectively. Person-years were calculated until age of CRC for cases (*PMS2* carriers with CRC, n = 125), and age at polypectomy, age of death, or last known age alive (whichever occurred first) for controls (*PMS2* carriers without CRC, n = 1, n = 1 and n = 381 respectively). The mean age was 52.5 for CRC cases and 51.0 for non-cases (Table 1). For a detailed description of the families including genotypes see Supplemental Tables 2a + 2b.

**Table 1:** Cohort description

	No CRC—controls (n = 383)	CRC—cases (n = 124)	All (n = 507)
Sex			
Male	133 (34%)	60 (48%)	193 (38%)
Female	250 (65%)	64 (52%)	314 (62%)
Age (CRC or censoring)			
Mean (s.d.)	51.0 (14.2)	52.5 (12.7)	
Range	25–88	27–88	
Index carrier			
Yes	38 (10%)	89 (72%)	127 (25%)
No, family member	345 (90%)	35 (28%)	380 (75%)
Other cancers (no. of carriers)			
Endometrial cancer^#^	30	9	39
Ovarian^#^	4	0	4
Duodenal cancer^#^	4	2	6
Breast^#^	10	3	13
Urothelial^#^	4	2	6
Esophagus	1	0	1
Leukemia	0	3	3
Testis	2	0	2
Prostate	1	1	2
Vagina	0	1	1
Mesothelioma	0	1	1

‘Index carrier’ means the first person to be tested. Incidence of cancer in the group of index carriers without CRC: 20 endometrial cancers, 4 ovarian cancers, 3 breast cancers, 3 cancers of the small intestine, 1 testis cancer and 1 carcinoid. Ten of these index carriers had not developed any cancer at the time of DNA diagnostics; they were tested because of polyps at an early age or because they had an (affected) deceased family member

^#^ Lynch syndrome associated cancer

### Hardy–Weinberg equilibrium

Two SNPs, rs1048943 (15q24.1) and rs4925386 (20q13), were not in Hardy Weinberg equilibrium (HWE). Violation of the HWE was present in both cases and controls and as this might be the result of a genotyping error, these SNPs were removed from the analysis. Ultimately, 24 SNPs were included in the final analysis.

### Risk of colorectal cancer

**Fig. 1:**
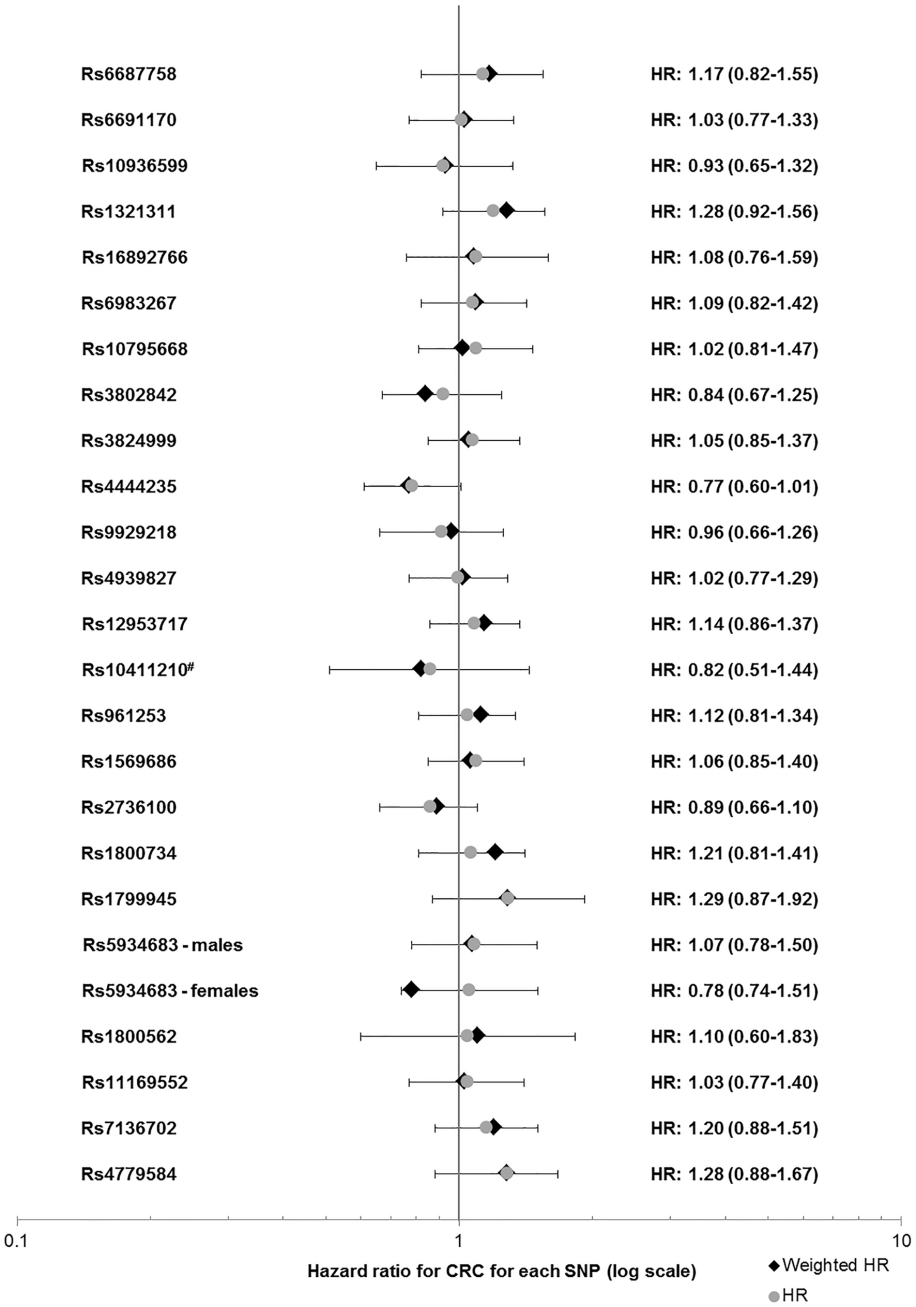
Forest plot of HRs for all SNPs. rs5934683 lies on the X chromosome and was therefore stratified for gender. *SNPs previously associated with increased risk in *MLH1* mutation carriers. ^#^Reference category: homozygous for risk allele (due to low number of homozygous carriers of the non-risk allele). *HR* Hazard ratio

**Fig. 2:**
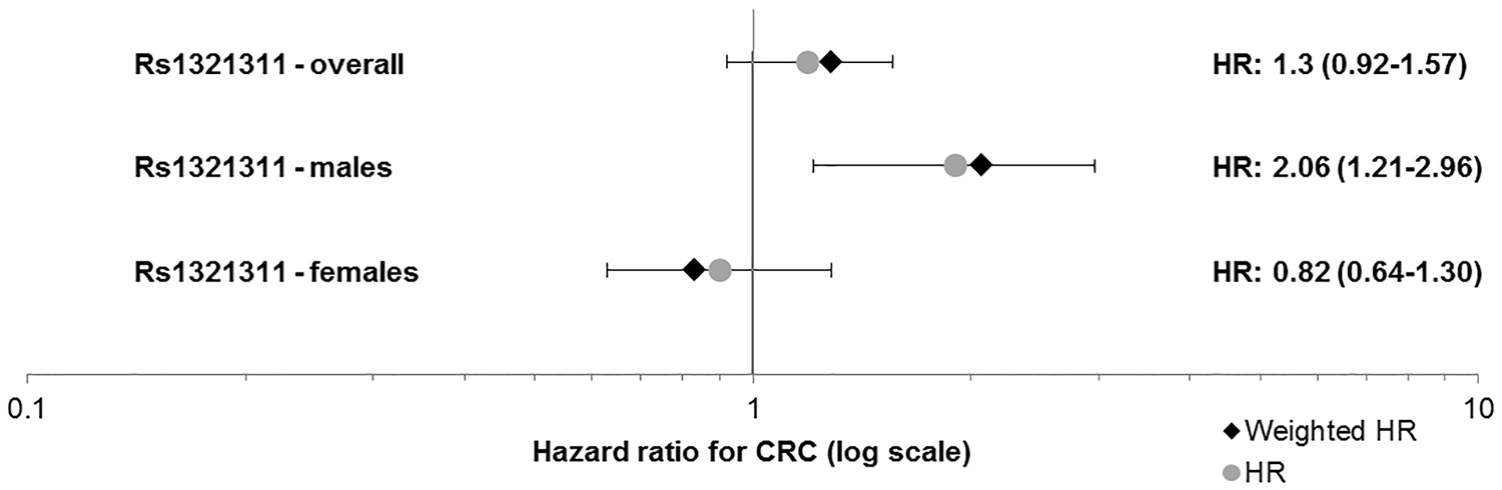
Forest plot of HRs for rs1321311. p = 0.005 for males. *HR* Hazard ratio

None of the SNPs individually showed a clear risk modifying effect (Fig. 1, Supplemental Table 1). There was a difference in HR between male and female *PMS2* carriers for rs1321311 (6p21.31), with an HR for the each additional A allele of 2.1 (95% CI 1.2–3.0, p = 0.005) and 0.83 (95% CI 0.63–1.28, p = 0.56) for males and females, respectively (Fig. 2: Forest plot, Supplemental Fig. 2: KM curve).

### Combination of rs3802842 and rs16892766

**Fig. 3:**
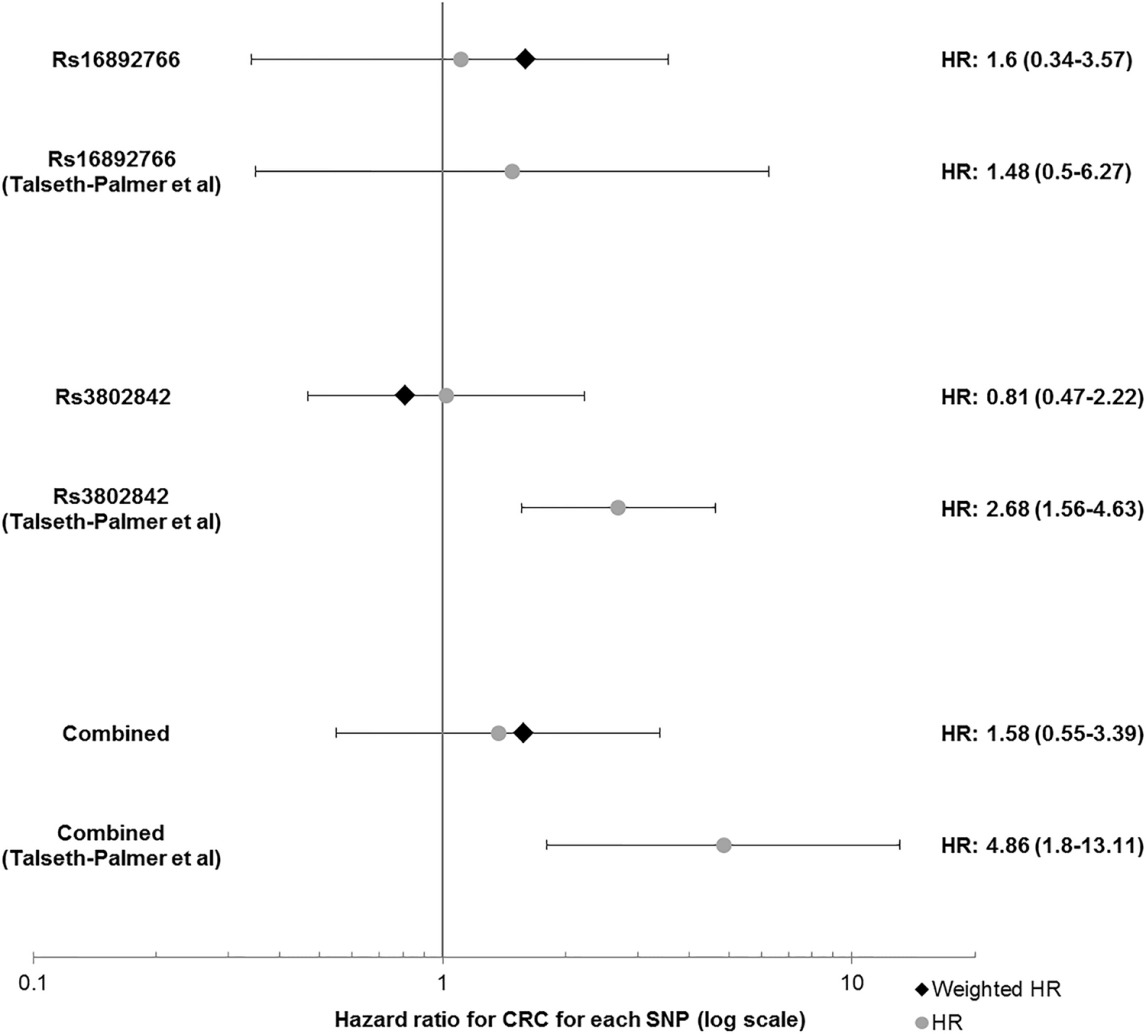
Forest plot of HRs for rs3802842 and rs16892766. For the combination of the two SNPs, the plotted HR represents a comparison for carriers of three vs. no risk alleles. *HR* Hazard ratio

A previous meta-analysis reported a significant pairwise effect on CRC risk of rs3802842 (11q23.1) and rs16892766 (8q23.3) in *MLH1* mutation carriers [6]. The HR in the additive model for this combination in our *PMS2* cohort was 0.95 (95% CI 0.80–1.25, p = 0.99). For carriers of more than three risk alleles the HR was 1.58 (95% CI 0.55–3.39) compared to patients with no risk alleles, see Fig. 3 for a comparison of previously publishes HRs and results from this study. The mean age at CRC diagnosis for 0, 1, and more than 1 risk alleles was 52.8; 52.9 and 50.4 respectively. The corresponding median ages were 54 [interquartile range (IQR): 43–62], 51 (IQR:43–63) and 47 (IQR:39–63). There was a statistically non-significant difference between the median age of CRC diagnosis between male and female carriers of two or more risk alleles, namely 53 (IQR:39–64, n = 7) and 43.5 years to age (IQR:38–63, n = 10, p = 0.56, Mann–Whitney test).

### Polygenic risk score

**Fig. 4:**
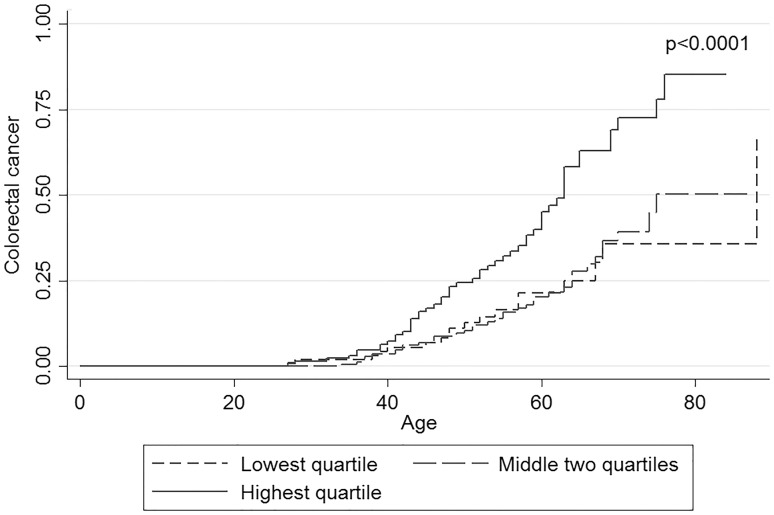
Kaplan Meier survival curve for PRS2. This plot compares curves for the lowest, the two middle and the highest quartile of the PRS. PRS2 is based on hazard ratios from the current study. *HR* Hazard ratio, *PRS* polygenic risk score

The polygenic risk score was calculated for 444 *PMS2* carriers with complete genotyping. The medians for PRS1 (meta-analysis derived ORs) were − 0.12 [interquartile range (IQR): − 0.48 to 0.30) for controls and − 0.03 (IQR: − 0.39 to 0.40) for CRC cases. The HRs for group 2 (second and third quartile) and 3 (fourth quartile) were 1.33 (95% CI 0.76–2.33) and 1.50 (95% CI 0.82–2.72) respectively (Table 2). The medians for PRS2 (based on HRs from our own data) were 0.30 (IQR: − 0.057 to 0.55) for controls and 0.51 (IQR: 0.068–0.75) for CRC cases. The corresponding HRs for group 2 and 3 were 1.05 (95% CI 0.59–1.89) and 2.62 (95% CI 1.49–4.60) respectively (Table 2). The KM curves for PRS1 and 2 are shown in and Supplemental Fig. 3 and Fig. 4, respectively. The difference between survival curves was highly significant for PRS2 (p < 0.0001). The optimism-corrected c statistic was only 0.52, indicating no discriminatory value.

**Table 2:** Polygenic risk scores

	PRS category	Controls	CRC	Expected events	HR	p for HR
PRS1	Median (IQR)	− 0.12 (− 0.48 to 0.30)	− 0.03 (− 0.39 to 0.40)			
	First quartile	84	22	28	Ref	0.41
	Second and third quartile	167	54	52	1.33 (0.76–2.33)	0.31
	Fourth quartile	84	33	28	1.50 (0.82–2.72)	0.19
PRS2	Median (IQR)	0.30 (− 0.057 to 0.55)	0.51 (0.068–0.75)			
	First quartile	84	18	26	Ref	< 0.0001
	Second and third quartile	165	38	53	1.05 (0.59–1.89)	0.86
	Fourth quartile	84	53	30	2.62 (1.49–4.60)	0.001

*PRS* polygenic risk score, *IQR* interquartile range

*PRS1* Weighted on odds ratios from general population, i.e. in sporadic CRC cases

*PRS2* Weighted on hazard ratios from this study

Log rank survival curves PRS1: p = 0.32

Log rank survival curves PRS2: p < 0.0001

## Discussion

*PMS2* carriers currently represent a relatively small proportion of LS patients. However, the number of *PMS2*-associated LS cases is expected to rise with the implementation of population-based screening protocols for all CRC below age 70. Identification of *PMS2* carriers has been challenging in the past due to difficulties in mutation analysis, a milder phenotype and many families not fulfilling clinical selection criteria [3, 9, 15–17]. Obtaining a better understanding of the specific *PMS2*-associated phenotype is particularly relevant, as it appears to differ markedly from phenotypes associated with other MMR mutations. Unfortunately, we were unable to confirm any risk modifying effects of rs3802842 (11q23.1) and rs16892766 (8q23.1), two SNPs previously shown to be associated with enhanced risk in *MLH1* mutation carriers [5, 6]. Studies in *MLH1* mutation carriers reported that a higher number of risk alleles in a carrier is associated with a younger onset of disease (28 years younger for 3 compared to 0 risk alleles) [6]. In our cohort, mean ages where 52.8 and 50.4 for 0 compared to more than 1 risk allele, respectively. As such, there seems to be no clinical utility of rs3802842 and rs16892766 in risk stratification for *PMS2* carriers.

Many studies on (genetic) modifiers in LS patients focus on *MLH1* and *MSH2*, or *MSH6* carriers, while *PMS2* is seldom analyzed. The only study to include *PMS2* carriers (n = 40) found that carriers of the G-alleles of rs10795668 (10p14) and rs9929218 (16q22.1) were at lower risk of CRC, a notable finding in that this is the opposite effect compared to sporadic CRC [7]. The authors conceded that their results should be confirmed in larger studies. As these findings have not been confirmed in our much larger cohort, we suggest that these previous findings may indeed have been false positives due to the small number of carriers included.

A relevant question is why our study did not confirm reported findings of previous studies of MMR carriers. One explanation might be that although patients have germline mutations in genes with similar functions, carriers are affected by genetic modifiers in different ways. Indeed, comparable studies in *BRCA1* or *BRCA2* mutation carriers have resulted in the identification of SNPs that clearly modify breast cancer risk. However, *BRCA2* carriers appear unaffected by SNPs that confer an increased breast cancer risk in *BRCA1* carriers, even though both genes play a role in homologous recombination [18]. This could also hold for MMR mutation carriers, as illustrated by the observation that while rs3802842 and rs16892766 may increase risk in *MLH1* carriers, they do not appear to have an effect in *MSH2* or *PMS2* carriers [5, 6]. Researchers should therefore concentrate on building cohorts large enough to analyze Lynch patients in a gene-stratified manner.

Gender stratification in our cohort led to the notable finding that male carriers of allele A at locus rs1321311 (6p21.31) show a per allele HR of 2.07 (95% CI 1.21–2.96), while the HR for females was 0.83 (95% CI 0.63–1.28). This SNP has been linked to the *CDKN1A* gene that encodes the p21 protein. p21 is involved in several (p53-independent) pathways as a tumor suppressor, although it also has oncogenic characteristics [19, 20]. Interestingly, down-regulation of p21 is inversely associated with MSI, the hallmark of Lynch-associated tumors. One study found that a larger proportion of Lynch-associated CRCs expressed p21 compared to sporadic CRCs (80 vs. 31%) [21]. However, a recent study reported on expressive Quantitative Trait Loci (eQTL) in colonic tissue based on data from the GTEx project portal (http://www.gtexportal.org/home/) and did not find a statistically significant effect of rs1321311 on CDKN1A expression in sigmoid and transverse colon tissue (p = 0.84 and p = 1.00 respectively) [22, 23]. It is also unclear why this effect only appears to be present in male *PMS2* carriers. Although no gender difference was noted by the meta-analysis that identified the SNP [19], it is possible that gender differences exist, as CRC risk in the general population and in Lynch patients is known to be higher in men compared to women [24, 25]. Indeed, mutations are more often found in males than females when assessed for Lynch syndrome [26]. Another possible explanation for this gender specific effect might lie in the effect of other risk modifiers. It is perceivable that other factors than SNPs have a stronger influence on (colorectal) cancer development in women, such as hormonal factors [27, 28]. Unfortunately data on hormone levels or other factors previously shown to modify cancer risk in LS such as medication use (e.g. aspirin) or environmental factors were unavailable for analysis and as such we were unable to correct for this [29–33]. Similarly, data on smoking and BMI were only available for a small proportion of carriers (n = 131, 26%). It should be emphasized that all results after gender stratification should be interpreted with caution because of small sample size and multiple testing. This could have led to false associations. Further studies are needed to validate these findings.

We also investigated the effect of the 24 SNPs on CRC risk in the *PMS2* cohort by means of a polygenic risk score (PRS). While there did not appear to be a significant effect of the PRS based on ORs from sporadic CRC cohorts, there was a difference in the cumulative incidence of CRC for *PMS2* carriers with a PRS2 (based on HRs in this study) in the highest quartile. Bootstrap validation however refuted this promising observation. Further studies are hence needed in other large cohorts.

There were some limitations to this study. Our study consisted exclusively of Dutch *PMS2* carriers and thus had a relatively homogeneous genetic makeup, implying that differences between our results and previous studies might be due to population-specific effects.

A second limitation might be that we did not correct for the specific mutation present in each family, mainly because in the majority of families the segregating *PMS2* mutation is rare or even unique. A previous study by our group did not identify such a correlation with CRC risk in *PMS2* carriers (Supplemental Tables 2a + 2b Table: for more details) [34].

Unfortunately, we were not able to validate our findings in an external cohort. To our best knowledge this is one of the largest *PMS2* cohorts currently collected, and bootstrap validation is a strong approach to assess discriminative ability of a prediction model [35]. Stratifying our cohort into a discovery and validation cohort was not a viable option as this would have resulted in a substantial decrease in power. Our study might already have been underpowered to detect weak associations. However, while such associations are interesting from a scientific point of view and may be relevant to tumorigenesis, they are not necessarily useful in clinical practice when the effect is small. For the two SNPs previously found to increase risk in *MLH1* mutation carriers, we had 60–80% power to detect an HR of 1.5, which we would consider clinically relevant. The previously reported HR in *MLH1* carriers for rs3802842 was 2.7, an HR for which we have ample power to detect (Supplemental Fig. 1).

Families with a segregating *PMS2* mutation show a high degree of phenotypic variability. We were not able to confirm the risk modifying effect of rs3802842 (11q23.1) and s16892766 (8q23.3), which were previously found to increase the risk in *MLH1*-associated LS. This, together with the established lower penetrance, raises the question of whether *PMS2*-associated LS should be considered a separate Lynch disease entity. Additional explanations for phenotypic variability that warrant greater exploration include gene-environment interactions and risk modification by other genetic variants.

## Electronic supplementary material

Below is the link to the electronic supplementary material.

**Supplemental Table 1: Overview of all tested SNPs, including gender stratification** Threshold for multiple testing: p < 0.0005. HR=Hazard Ratio. OR=Odds Ratio. 95%CI= 95% confidence interval. MAF=minor allele frequency. (w)HR: (weighted) Hazard Ratio. #This meta-analysis uses homozygotes for the major allele as a reference category. **Supplemental Table 2a: Overview of germline PMS2 mutations in the cohort**. Each row describes 1 family, **Supplemental Table 2b: Most frequent germline PMS2 mutations in the cohort**. (DOCX 93 KB)

**Supplemental File 1: Supplemental method – statistical analysis** (DOCX 31 KB)

**Supplemental Figure 1: Post hoc power analysis**. Purple dashed lines indicate the hazard ratios previously reported for rs3802842 (11q23.1) and rs16892766 (8q23.3). (TIF 62335 KB)

**Supplemental Figure 2: Kaplan Meier survival curve**. Note: 0=homozygous for non-risk allele. 1=Heterozygous/homozygous for the risk allele. p=0.0043. (TIF 2126 KB)

**Supplemental Figure 3: Kaplan Meier survival curve for PRS1**. Note: This plot compares curves for the lowest, the two middle and the highest quartile of the PRS. PRS1 is based on meta-analysis derived ORs in sporadic colorectal cancer cohorts. PRS: polygenic risk score. (TIF 2126 KB)
