# Supplementary material for: SNP association study in PMS2-associated Lynch syndrome
Source: Fam Cancer. 2017 Nov 17;17(4):507–15. doi: 10.1007/s10689-017-0061-3 (PMC6182583; doi:10.1007/s10689-017-0061-3)
Supplement: Supplementary file 2 — Supplemental File 1: Supplemental method – statistical analysis (DOCX 31 KB) [file 10689_2017_61_MOESM2_ESM.docx]

**Supplemental method – statistical analysis**

*Follow-up and data-collection:*

For the vast majority of pre-symptomatically tested family members, i.e. controls, last known age was age at DNA diagnosis and thus – in general - age at start of colonoscopic surveillance. This is due to the fact that carriers were ascertained through genetic centres, which made us unable to collect follow-up data, as subsequent surveillance was done at the gastroenterology and/or gynaecology departments.

*Ascertainment bias & weighted analysis:*

All subjects included in this study were derived from family cancer clinics and were therefore not randomly selected with respect to their phenotype. In other words they were selected based on the occurrence of cancer at a young age or due to several family members being affected. Therefore, these carriers usually belong to high-risk families, ascertained as a consequence of their relatively severe phenotype. It is likely that other factors than the germline mutation in PMS2 and the SNPs currently investigated may play a role in the phenotype variability, such as lifestyle or other genetic factors.^1^

Weights were calculated based on incidence rates in the Dutch population. HRs based on a proportion of this cohort have been previously reported and were used to determine age stratum (5 year) specific weights.^3^ All calculated weights for cases were smaller than 1, effectively down-weighting cases compared with controls. It is important to note that for hypothesis testing the unweighted p-value and confidence interval are reliable.^12^ We therefore report the weighted HRs with both the p-values and the 95% confidence intervals from the unweighted analysis. Both unweighted and weighted HRs are listed in the tables.

*Polygenic risk score:*

PRS1 and 2 included all 24 SNPs that were found to be in Hardy Weinberg equilibrium, however two SNPs in the HFE gene were not taken into account in the PRS1 calculation, because to our knowledge there were no ORs from meta-analysis reported in current literature. The PRS was calculated as previously described^16^, by using the following formula for PRS1 and PRS 2 respectively:

$$\sum_{i=1}^{n} a_{i}\log{OR}_{i}$$

$$\sum_{i=1}^{n} a_{i}\log{HR}_{i}$$

where n is the number of SNPs, a is the number of risk alleles for each SNP and the OR the meta-analysis derived OR (supplementary table 1)^4^ or the HR from the current study for each SNP for PRS1 and PRS 2 respectively. *PMS2* carriers were categorized into four groups of equal sizes based on the quartiles in the control group.

*Multiple testing:*

Correction for multiple testing was done by calculating the corrected overall critical p-value for all performed tests (four per SNP, including gender stratification, four PRS tests and the combination of rs3802842 and rs16892766) using the Bonferroni method. This leads to a p value 0.05/101= 0.0005.

*Other:*

Mean age at CRC development was examined using one-way Analysis of Variance (ANOVA).
