# Supplementary figures and images for: SNP association study in PMS2-associated Lynch syndrome
Source: Fam Cancer. 2017 Nov 17;17(4):507–15. doi: 10.1007/s10689-017-0061-3 (PMC6182583; doi:10.1007/s10689-017-0061-3)

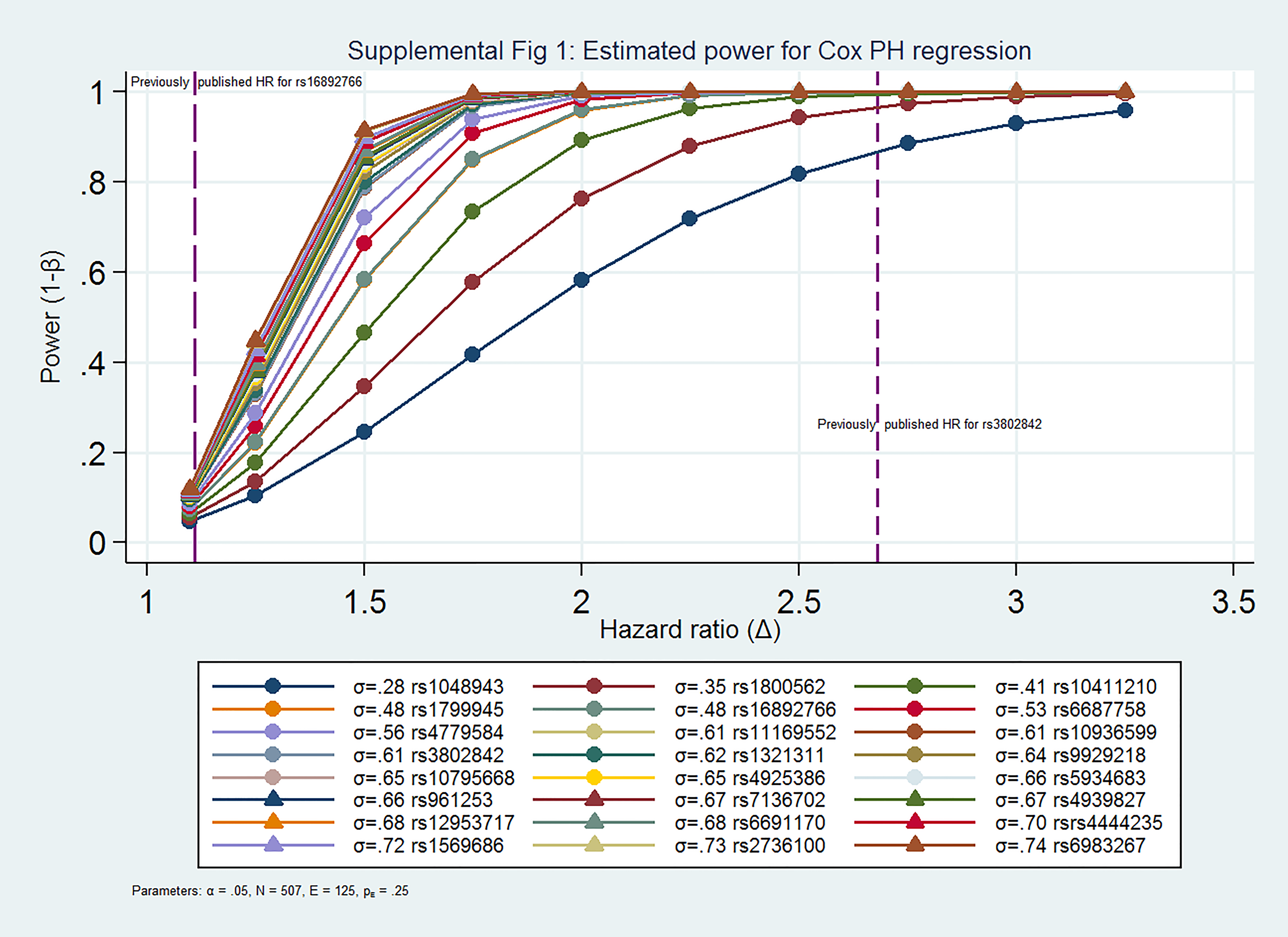

Supplement: Supplementary file 3 — Supplemental Figure 1: Post hoc power analysis. Purple dashed lines indicate the hazard ratios previously reported for rs3802842 (11q23.1) and rs16892766 (8q23.3). (TIF 62335 KB) [file 10689_2017_61_MOESM3_ESM.tif]

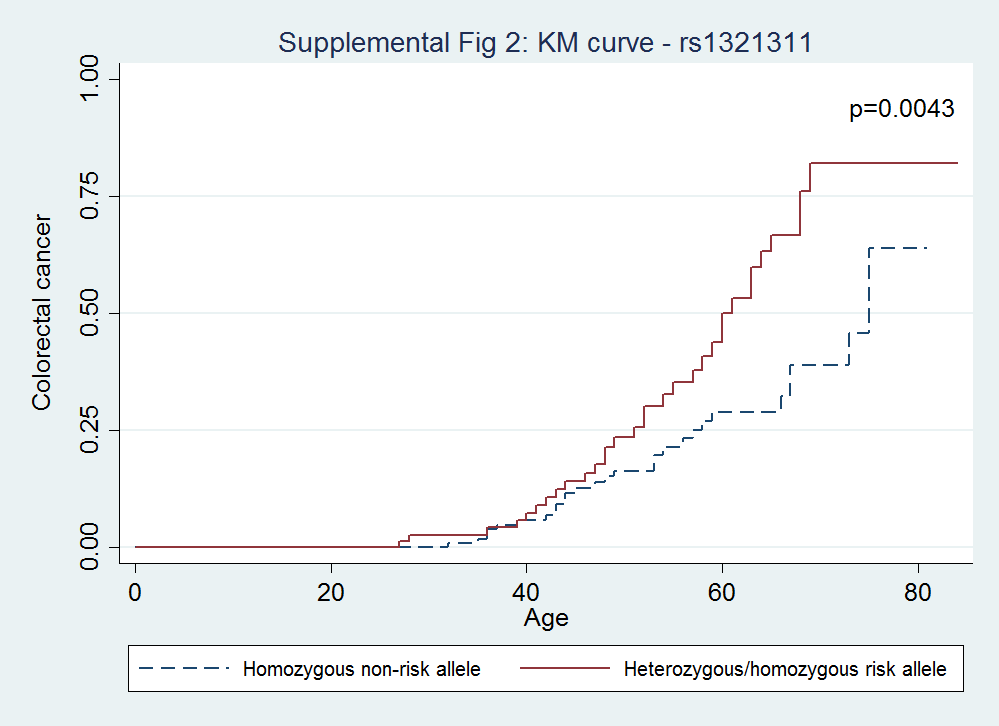

Supplement: Supplementary file 4 — Supplemental Figure 2: Kaplan Meier survival curve. Note: 0=homozygous for non-risk allele. 1=Heterozygous/homozygous for the risk allele. p=0.0043. (TIF 2126 KB) [file 10689_2017_61_MOESM4_ESM.tif]

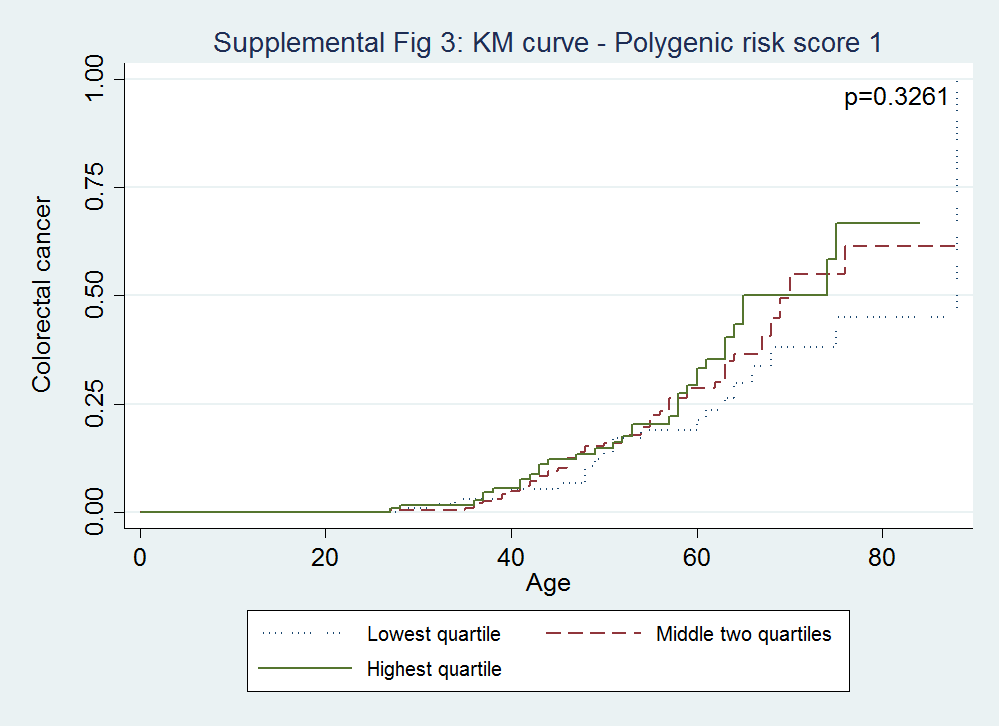

Supplement: Supplementary file 5 — Supplemental Figure 3: Kaplan Meier survival curve for PRS1. Note: This plot compares curves for the lowest, the two middle and the highest quartile of the PRS. PRS1 is based on meta-analysis derived ORs in sporadic colorectal cancer cohorts. PRS: polygenic risk score. (TIF 2126 KB) [file 10689_2017_61_MOESM5_ESM.tif]
